# Suppurative Pericarditis

**Published:** 1895-12

**Authors:** C. L. Norsworthy

**Affiliations:** While an Interne of the Charity Hospital of New Orleans, La., now of Houston, Texas


For Texas Medical Journal.

### SUPPURATIVE PERICARDITIS.

### Report of a Case, and Some Important Objective Signs for Diagnosing Pericardial Effusion.

BY C. L. NORSWORTHY, M. D.,
While an Interne of the Charity Hospital of New Orleans, La., now of Houston, Texas.

ED G., a colored male, 18 years of age, was a coal wheeler by occupation. Family history of no importance. He had been exposed to cold and wet weather for several days and nights previous to time of taking sick. Patient's health had been perfect until twenty-one days ago, when he was taken sick with left lobar pneumonia, I suppose, from his description of it, for which he had received no attention whatever. No traceable suspicion of tuberculosis nor rheumatism. I saw the patient on January 31st, when he complained of a dull oppressive pain in his precordial region which was increased by pressure.

On inspection, his general appearance indicated that of an inane and a very badly neglected man. His skin was very cold, dry and scaly, eyelids were somewhat drooped and conjunctivæ very dry, as if all lachrymal secretions were arrested. Tongue was anemic, small, dry and heavily coated. Pulse was so rapid, small and feeble until I could not count it with any accuracy, and was very irregular and intermittent. Temperature was 101°; respiration was 36 per minute. The patient had a dry, hacking and iritable cough. Bowels were acting normally.

On inspecting the chest the left was found to be rather prominent, the third, fourth and fifth intercostal spaces were almost obliterated. The cardiac pulsation was entirely invisible and the respiratory expansion almost so. Right side normal.

On palpation anteriorily the cardiac impulse was scarcely perceptible, and the vocal fremitus of the left side was entirely absent. Posteriorily neither the cardiac impulse nor the vocal fremitus could be detected on the left side.

Neither mensuration nor succussion was practiced.

By percussion anteriorily the precordial dullness was found to extend from the first intercostal space down to the seventh costal cartilage, and laterally from one inch to the right of the sternum to near the center of the axillary. Posterior percussion revealed complete dullness over the left infra, and slight dullness over the left supra scapular regions. Right side was thought to be somewhat hyper-resonant, both anteriorily and posteriorily.

While auscultating anteriorily nothing could be heard over the left infra clavicular and mammary regions except the very faint heart sounds, which could be heard most distinct about the fourth rib and to the left of the nipple line. Surrounding the area of the precordial dullness could be heard very rough friction sounds. Posteriorily nothing could be heard over the left infra scapular region except rough friction sounds with very faint vocal resonance.

I diagnosed the case pericardial effusion and pluerisy with effusion resulting from left lobar pneumonia.

Treatment consisted of hot turpentine stupes over the entire left chest which was changed every hour or two and the hypodermic use of strychniæ sulphatis, atropiæ sulphatis, nitro glycerine and tr. digitalis. The patient was nourished with strong milk punches and Ducros elixir, each every three hours.

After the continued use of strychniæ, the nourishment and stupes and the alternate use of atropiæ and nitro glycerine for forty-two hours the pulse numbered 120, but was still very weak, small, irregular and intermittent. Temperature was 98, and respiration 46 per minute.

After the continued use of strychniæ, the nourishment and stupes and the alternate use of nitro glycerine and tr. digitalis for thirty-six hours more, pulse numbered 110, was a little stronger but still very small, irregular and intermittent.

The same treatment was kept up until death, which was three days later, during which time the pulse ranged from 90 to 110, respiration from 24 to 36 per minute, and temperature from 97° to 99°. The patient died suddenly. About two or three hours before death his temperature fell to 95 3-5°.

I held the autopsy the same day of death and found the pericardium to be much distended, very tense, and contained about 1½ pint of very thick and creamy pus, the odor of which was sweetish and somewhat nauseating. The lining pericardium was nothing more than a cheesy degenerated membrane and resembled very much a bruised honey comb. Heart was very anemic, small and firm, its walls were thickened and indurated, the ventricles were very small, auricles of medium size, and the endocardium was negative and free from atheroma.

Left lung was entirely collapsed, and the left plueral cavity was almost taken up by the distended pericardium.

There was considerable pleurisy and some adhesions between the pericardium and pleural membranes with a small quantity of pleural effusion. Right lung normal.

Some of the most prominent symptoms presented by three cases of pericardial effusion under my observation, one being more acute than the other two, with one death. The patients seemed to prefer quietude. They remained in their most comfortable position and appeared not to be cognizant of their surroundings. Their facial expression indicated suppression or as if they were in deep thought. The facial expression of the more acute case was that of anxiety and pain. The respirations were short and hurried, ranging from 26 to 40 per minute.

The lachrymal secretions were very much diminished. The patients preferred a partial sitting posture, leaning a little forward, while an increase of their leaning posture would cause a feeling of oppression and a sinking pain in the precordial region.

Their pulse was very small, irregular and intermittent, and ranged from 90 to 120 per minute. The pulse of the more acute case at times was dicrotic; he also suffered some from a pain in the left brachial region ranging down the arm. Each case presented more or less of a dry irritable cough. I found that pressure over the precordial region or by pressing the left lobe of the liver upwards would cause a sinking sensation, a sensation which they they described as if they were going to faint. Their temperature ranged from 97 2-5 to 102°, but was oftener below normal than above, except in the more acute case. There were no regular morning remissions nor evening exacerbations but the charts presented a rather zigzag appearance.

Hiccough was a persistent symptom in two cases. For a day or so previous to death the patient was partially comatosed at times. The temperature fell below normal shortly before death, which took place suddenly. The post mortem of the dead body corroborated my diagnosis.

